# Quality of life outcomes in colorectal cancer survivors: insights from an analytical study at a tertiary cancer center-Qatar

**DOI:** 10.3389/fonc.2025.1730212

**Published:** 2026-02-11

**Authors:** Nada Adli, Mohammed Adil Arbab Ahmed, Nahlah AlMesbah, Nagah Selim, Mohamed Abdelsalam Elimam Ibrahim, Sujood Abdulwakeel Musa Awadelseed, Mohamed Iheb Bougmiza, Hadi Mohamad Abu Rasheed, Kakil Rasul

**Affiliations:** 1 Community and Preventive Medicine Specialist, Preventive Medicine Division, Department of Internal Medicine, Hamad Medical Corporation (HMC), Doha, Qatar; 2 Qatar Cancer Society, Doha, Qatar; 3 Community and Preventive Medicine Department, Primary Health Care Corporation (PHCC), Doha, Qatar; 4 Professor of Public Health and Preventive Medicine, Cairo University, Ciro, Egypt; 5 Community and Preventive Medicine Department, Hamad Medical Corporation (HMC), Doha, Qatar; 6 College of Medicine, Qatar University, Doha, Qatar; 7 Professor of Community Medicine, Sousse University, Sousse, Tunisia; 8 Oncology Department, National Center for Cancer Care and Research, Hamad Medical Corporation (HMC), Doha, Qatar; 9 Associate Professor in Clinical Medicine at Weill Cornell Medicine, Doha, Qatar

**Keywords:** Quality of Life, colorectal cancer, predictors, adults, Qatar

## Abstract

**Background:**

Colorectal cancer (CRC) is a major public health concern that affects patients’ quality of life (QoL) and imposes challenges on families and healthcare systems.

**Objectives:**

To assess QoL and its determinants among CRC patients in Qatar and identify sociodemographic and clinical factors influencing QoL in 2023.

**Methods:**

An analytical cross-sectional study was conducted between July and December 2023. From the National Center for Cancer Care and Research (NCCCR) registry, 456 CRC patients were screened, and 169 eligible participants were included. QoL was evaluated using the EORTC QLQ-C30 and the CRC-specific QLQ-CR29 tools.

**Results:**

Most patients were aged ≥45 years (80.5%), non-Qatari (79.9%), and married (88.8%). Hypertension (39.1%) and diabetes (31.4%) were the most common comorbidities. Adenocarcinoma was predominant (90.5%), with most cases diagnosed at advanced stages (III–IV, 74.8%) and metastasis in 71.6%. The sigmoid colon was the most common site (34.9%), and combined surgery and chemotherapy were the main treatments (69.2%). Global QoL was moderate to high (70.4 ± 18.5), with fatigue (23.9 ± 26.8) and financial difficulties (28.9 ± 40.4) as the most reported concerns. Functional QoL was higher among those with sufficient income (+12.5 points, p < 0.01) and longer time since diagnosis (+0.06 points/month, p < 0.05), but lower among those with higher education (–5.0 points, p = 0.05) or alcohol use (–11.2 points, p = 0.05).

**Conclusions:**

Despite advanced disease stages, CRC patients in Qatar reported good QoL. Socioeconomic status significantly influenced outcomes, highlighting the need for integrated psychosocial and financial support.

## 1 Introduction

Cancer remains one of the leading causes of morbidity and mortality worldwide, responsible for nearly 10 million deaths and an estimated 19.3 million new cases in 2020 (1)Among the various malignancies, colorectal cancer (CRC) arising from the epithelial lining of the colon and/or rectum is one of the most common. Globally, CRC ranks third in terms of incidence, with 1.93 million new cases in 2020 (10.6% in males and 9.6% in females across all age groups), and second in cancer-related mortality, causing approximately 916,000 deaths in the same year (2).

The global burden of CRC continues to rise, and projections suggest an alarming 60% increase by 2030, reaching over 2.2 million new cases and 1.1 million deaths (3). CRC poses not only a clinical challenge but also a significant public health concern due to its substantial impact on patients’ health-related quality of life (HR-QoL). Advances in screening, chemotherapeutics, and surgical techniques have contributed to improved survival rates (4); however, these clinical gains must also be evaluated in terms of patient-reported outcomes, particularly QoL, which encompasses physical, psychological, social, and functional dimensions (5, 6).

Quality of life is now recognized as a critical endpoint in cancer care and research (5). Studies indicate that CRC patients frequently experience a spectrum of disease- and treatment-related complications, including pain, fatigue, depression, anxiety, sleep disturbances, and gastrointestinal dysfunction, all of which adversely affect HR-QoL (7, 8). Furthermore, QoL outcomes vary substantially across populations, influenced by cultural, socioeconomic, and healthcare system differences. For example, research has demonstrated that CRC patients in some regions continue to report poor QoL in domains such as physical, social, clinical, and financial functioning (7).

Multiple sociodemographic and clinical variables have been shown to influence HR-QoL in CRC patients. Factors such as age, sex, marital status, education level, financial stability, living conditions, and smoking history are frequently associated with QoL outcomes. Clinical determinants include disease stage, time since diagnosis, type and duration of treatment, hospital stay, treatment response, presence of metastases, and comorbidities also play a significant role. For instance, a study conducted in Saudi Arabia among 106 CRC patients revealed that employment status and tumor location were strongly associated with declines across multiple QoL domains (9–13). A recent systematic review further highlighted that cancer-related symptoms beyond gastrointestinal manifestations, including pain, fatigue, depression, anxiety, dyspnea, and insomnia, significantly reduce HR-QoL and may vary according to disease stage and treatment modality (8).

In Qatar, CRC is the most common malignancy among men (15.7% of all new cancers) and the second most common among women (7.5%), following breast cancer. In 2017, 178 new cases of CRC were registered: 115 (66.6%) males and 63 (35.4%) females. The majority of patients survived, with only 2.9% mortality at the time of reporting (14). The peak incidence was observed in men aged 60–64 years and women aged 55–59 years, with the youngest case diagnosed in a 20-year-old male. Histologically, adenocarcinoma, not otherwise specified (NOS), was the predominant type (75.8%), followed by neuroendocrine carcinoma (7.9%) and malignant neoplasm (5.6%) (14). Staging data were available for 28% of patients, with 40% presenting at stage IV, 27% at stage III, and 22% at stage II. Treatment information was reported for 75.8% of cases, with surgery (31.9%), chemotherapy (8.9%), radiotherapy (2.2%), and multimodal therapies being the most common approaches (14). Recognizing the high incidence and mortality rates of CRC, Qatar has implemented a national screening program through the Primary Health Care Corporation (PHCC), alongside extensive community-based health education campaigns to raise awareness about risk factors, prevention, and the importance of early detection (15).

Despite these initiatives, a major research gap persists regarding the broader impact of CRC on patients’ quality of life in Qatar. While clinical outcomes such as survival are relatively well documented, little is known about how CRC affects the physical, psychological, social, and functional well-being of Qatari patients. Given that QoL is an essential outcome for evaluating the full impact of CRC on individuals, families, and the healthcare system, addressing this gap is crucial. The present study therefore seeks to assess the QoL of colorectal cancer patients in Qatar and to identify sociodemographic and other factors associated with QoL outcomes.

## 2 Methods

### 2.1 Study design and setting

An analytical cross-sectional study was conducted in Qatar from July to December 2023 among patients diagnosed with colorectal cancer who were receiving regular follow-up care at the oncology clinic. Data was obtained from the National Center for Cancer Care and Research (NCCCR) registry at Hamad Medical Corporation (HMC). NCCCR is the sole specialized cancer hospital in Qatar, providing comprehensive oncology services, including advanced therapies, hematology care, bone marrow transplantation, and palliative services, while surgical oncology is delivered in collaboration with Hamad General Hospital (16).

### 2.2 Study population and procedure

After approval from the Institutional Review Board of Hamad Medical Corporation (MRC-01–22-536), a list of colorectal cancer (CRC) patients (N = 456) was obtained from the National Center for Cancer Care and Research (NCCCR) registry and screened to identify eligible participants according to the study criteria. Eligible participants were adults aged 18 years or older, of any gender or nationality, who had been diagnosed with CRC, were receiving treatment at the NCCCR, and were able to communicate in either Arabic or English. Patients with cognitive or psychiatric disorders that could impair communication or influence HRQOL responses were excluded.

Verbal informed consent was obtained, and participants were invited to complete a telephone interview lasting approximately 20–30 minutes. Clinical data, including date of diagnosis, tumor site, and metastatic status, were extracted from medical records by trained research team members, while patient-reported outcomes were collected using a structured, multi-component questionnaire. Participation was voluntary, and participants retained the right to withdraw at any stage without consequence.

### 2.3 Questionnaire development and validation process

This part was developed and constructed by the author after an extensive literature review. It contained 15 items designed to collect information on socio-demographic, health-related characteristics and cancer related variables. The face validity of the questionnaire was established and ensured by consultation with the preventive medicine faculty and oncology experts in the field. Translation validity was established by translating the English version into Arabic by two native Arabic speakers and then translating it back into English to ensure consistency. All authors then agreed upon the final version. Subsequently, the questionnaire was piloted on a convenient sample of 10 patients to assess its clarity, comprehensibility, and appropriateness.

### 2.4 Data collection tools and variables

#### 2.4.1 Dependent variables

The primary outcome was the quality of life (QoL) of colorectal cancer (CRC) patients in Qatar, assessed using the validated EORTC QLQ-C30 and QLQ-CR29 instruments in both Arabic and English languages (17, 18). The QLQ-C30 includes 30 items covering five functional scales (physical, role, cognitive, emotional, social), three symptom scales (fatigue, pain, nausea/vomiting), a global health status/QoL scale, and six single-item scales assessing dyspnea, insomnia, appetite loss, constipation, diarrhea, and financial impact. Items are rated on a four-point Likert scale (1 = “not at all” to 4 = “very much”), except for the global health status scale (1 = “very poor” to 7 = “excellent”), with scores transformed to 0–100. Higher scores indicate better functioning and QoL (19). The QLQ-CR29 comprises 29 items, including four multi-item scales assessing urinary frequency, fecal seepage, stool consistency, and body image, alongside single items addressing other post-treatment issues; it distinguishes between clinically distinct patient groups and demonstrates reproducible scores over time in stable patients. Higher scores in symptom domains reflect greater symptom burden and poorer QoL (17).

#### 2.4.2 Independent variables

Independent variables comprise sociodemographic, health-related, and cancer-specific characteristics. Specifically, sociodemographic factors included age, gender, nationality, marital status, educational level, employment status, monthly income (insufficient, partially sufficient, or sufficient), and living conditions (alone, with family, with a caregiver, or in a long-term care facility). In addition, health-related variables encompassed the presence and type of comorbidities, such as hypertension, diabetes mellitus, cardiovascular disease, chronic respiratory disease, cancer, chronic kidney or liver disease, immune disorders, and psychological conditions including anxiety or depression. Furthermore, cancer-specific variables included cancer type (e.g., adenocarcinoma), disease stage (I–IV), tumor location (cecum, ascending colon, hepatic flexure, transverse colon, splenic flexure, descending colon, sigmoid colon, rectum, rectosigmoid, or anus), time since diagnosis, presence of metastasis (yes/no), and type of treatment received, including surgery, chemotherapy, radiotherapy, hormonotherapy, bone marrow transplantation, or combination therapy.

### 2.5 Ethical statement

This study was approved by the Medical Research Centre Ethical Committee (MRC-IRB); under protocol number (MRC-01-22-536). Verbal consent was obtained from each participant before the interview. The study was conducted in full conformance with the principles of the “Declaration of Helsinki” and Good Clinical Practice.

### 2.6 Data analysis

The database was constructed and analyzed using the Statistical Package for Social Sciences (SPSS)™ Software Version 25, R version 4.2.1 and RStudio 2023.09.0 Build 463. Descriptive analysis was performed for the characteristics of the participants and data was presented as frequencies and percentages for categorical variables. Additionally, the normality distribution of the dataset was assessed using the Kolmogorov-Smirnov test. The utilization of either the mean ± standard deviation (S.D) or the median ± Inter-Quartile Range (IQR) depended on the p-value obtained from the test. Non-parametric bootstrapped regression was also used to identify the effect of the sociodemographic and the clinical variables on patients’ quality of life, and to calculate the beta coefficients with the 95% Confidence Interval (CI). P-values ¾ 0.05 (two tailed) were considered statistically significant.

## 3 Results

### 3.1 Sample characteristics

**Figure 1:**
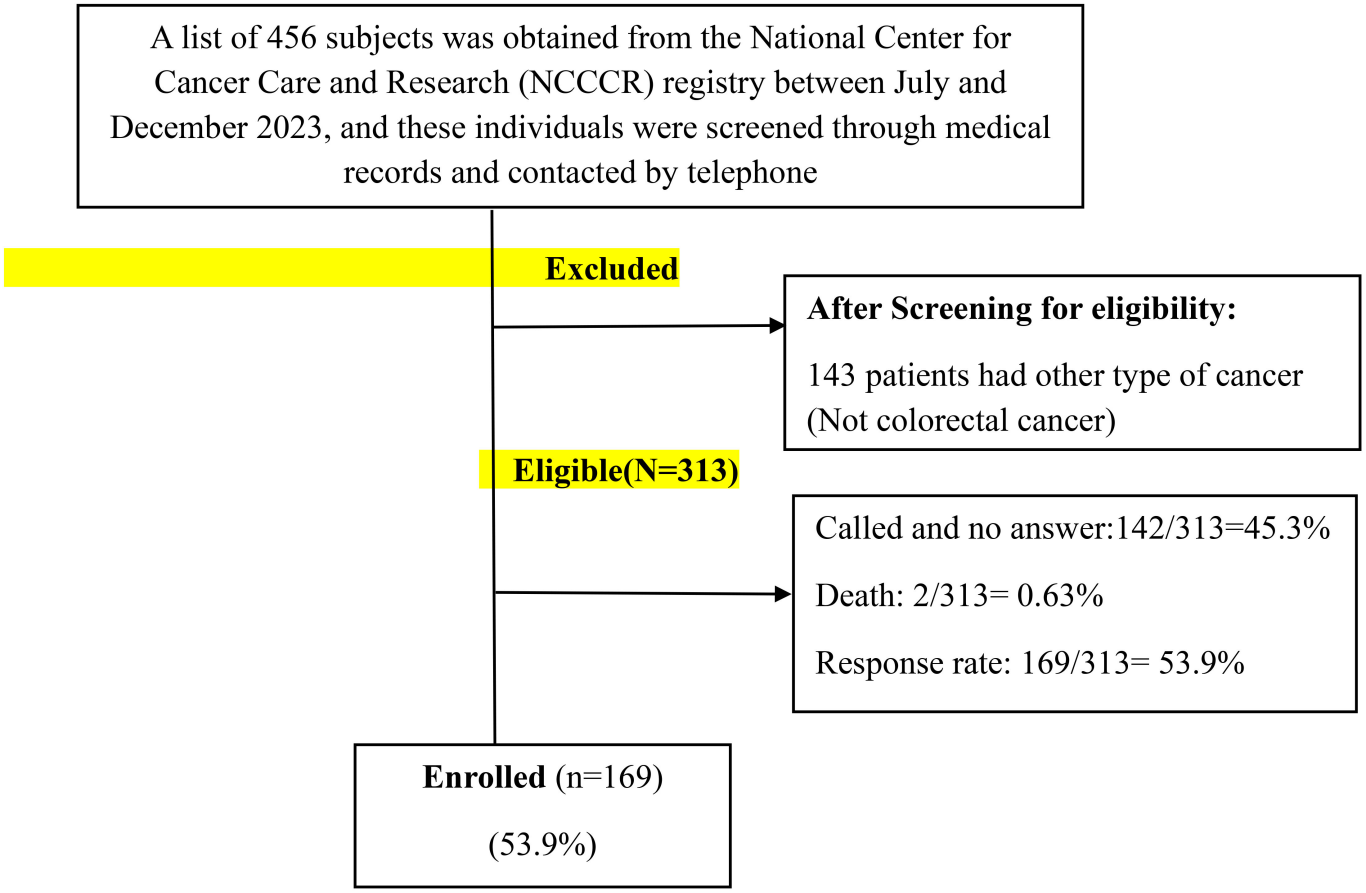
Flow chart of the study recruitment of the participants (n = 169).

During the data collection period, 456 individuals aged 18 years and above were initially identified, and their medical records were screened for potential participation. Upon review, 143 individuals were found to have diagnoses other than colorectal cancer and were therefore excluded. The remaining 313 eligible patients were subsequently contacted via telephone, yielding a response rate of 53.9%, as illustrated in **Figure 1**.

#### 3.1.1 Sociodemographic, and background characteristics of the study populations

**Table 1** presents the sociodemographic and background characteristics of colorectal cancer (CRC) participants in Qatar during 2023. Most CRC patients were aged 45 years and older (80.5%), non-Qatari (79.9%), married (88.8%), highly educated (66.4%), living with their family (80.7%), and had sufficient monthly income (48.1%). Furthermore, 45% of CRC patients had a history of other chronic diseases, including hypertension (39.1%) and diabetes (31.4%). Moreover, 11.2% of colorectal cancer patients reported being smokers, while 7.7% reported alcohol consumption.

**Table 1:** Sociodemographic and background characteristics of colorectal cancer participants in Qatar during 2023, (N = 169).

Variable	Frequency (n)	Percentage (%)
Age		
25-34	4	2.4
35-44	29	17.2
45-54	51	30.2
55-64	46	27.2
65 and above	39	23.1
Gender		
Male	85	50.3
Female	84	49.7
Nationality		
Qatari	34	20.1
Non-Qatari	135	79.9
Marital status		
Married	150	88.8
Not married	19	11.2
Educational level (n=152)		
Up to secondary school	51	33.6
University and higher education	101	66.4
Employment status (n=163)		
Not employed	66	40.5
Employed	97	59.5
Monthly income (n=131)		
Insufficient	38	29
Partially sufficient	30	22.9
Sufficient	63	48.1
Living condition (n=161)		
Live alone	30	18.6
Live with family	130	80.7
Living with care giver	1	0.6
History of chronic disease		
Yes	76	45
No	93	55
Hypertension		
Yes	66	39.1
No	103	60.9
Diabetes		
Yes	53	31.4
No	116	68.6
Smoking status		
Non-smoker	150	88.8
Smoker	19	11.2
Alcoholic		
Yes	13	7.7
No	156	92.3

**Table 2** outlines the clinical characteristics of colorectal cancer (CRC) patients in Qatar during 2023. Adenocarcinoma was the most prevalent histological type among CRC patients, accounting for 90.5% of cases. Most patients (74.8%) presented with advanced stages of cancer (stages 3 and 4), and a significant proportion (71.6%) exhibited metastasis to other locations.

**Table 2:** Cancer related-clinical characteristic among colorectal cancer patients in Qatar during 2023, (N = 169).

Variable	Frequency (n)	Percentage (%)
Type of cancer		
Adenocarcinoma	153	90.5
Others	16	9.5
Stage of cancer (n=167)		
Stage 1	11	6.6
Stage 2	31	18.6
Stage 3	68	40.7
Stage 4	57	34.1
Metastasis		
Yes	121	71.6
No	48	28.8

#### 3.1.2 Cancer-related-clinical characteristics of the study populations

**Figure 2:**
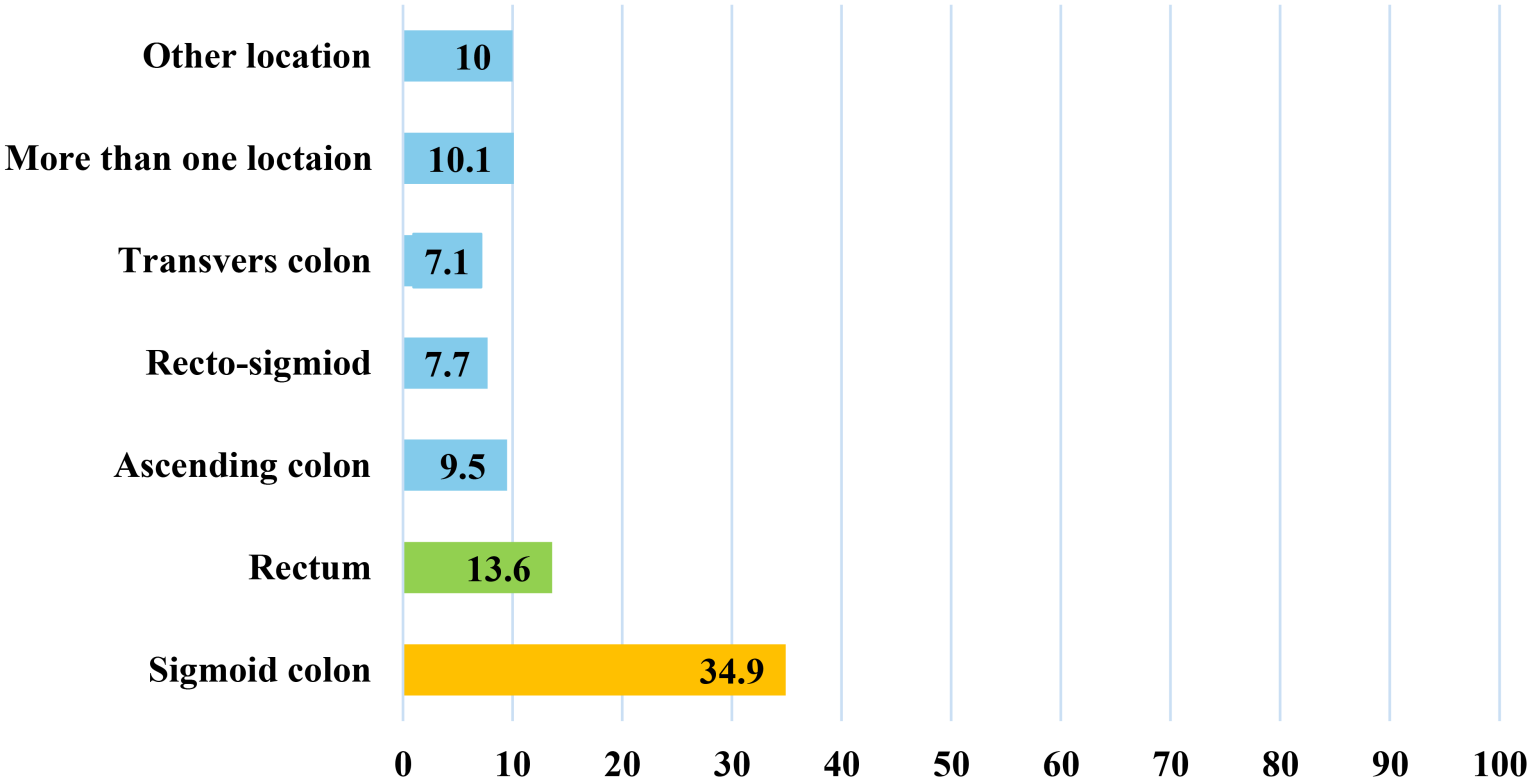
Frequency distribution of the tumor location among colorectal cancer patients in Qatar during 2023, (N = 169).

**Figure 2** illustrates the frequency distribution of tumor locations among colorectal cancer (CRC) patients. The sigmoid colon emerges as the predominant tumor location, accounting for 34.9% of cases, followed by the rectum (13.6%), ascending colon (9.5%), and recto-sigmoid colon (7.7%).

**Figure 3:**
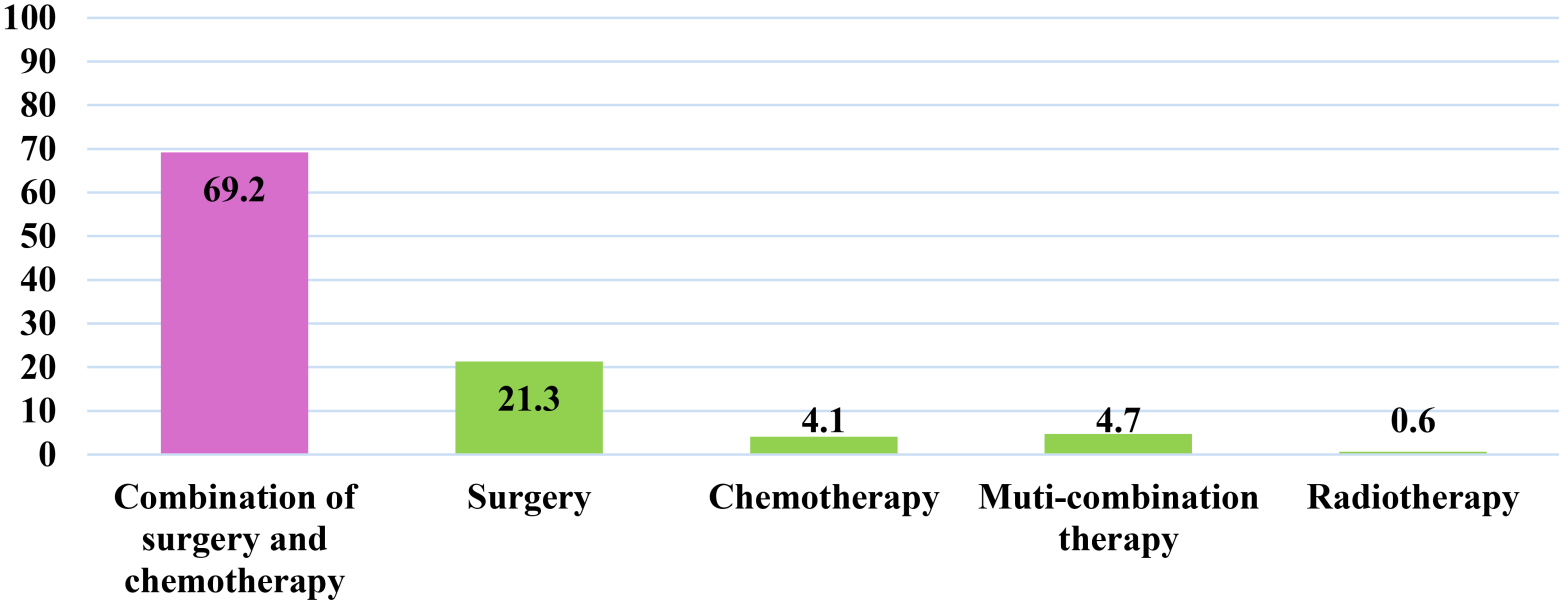
Frequency distribution of the types of treatment among colorectal cancer patients in Qatar during 2023, (N = 169).

Most of the colorectal cancer (CRC) patients (69.2%) underwent combined treatment involving surgery and chemotherapy. However, a notable proportion received monotherapy, with 21.3% undergoing surgery alone, 4.1% receiving chemotherapy alone, and 0.6% undergoing radiotherapy alone, as depicted in **Figure 3**.

#### 3.1.3 Quality of life among colorectal cancer patients in Qatar

**Table 3**; **Figure 4** present the distribution of quality of life (QoL) scores among colorectal cancer patients in Qatar, as measured by the EORTC QLQ-C30 and QLQ-CR29 instruments. The results indicate that patients reported a relatively high overall QoL, with a mean Global Health Status/QoL score of 70.4 (SD = 18.5) on the QLQ-C30. Functional outcomes were also favorable, with mean scores of 80.6 (SD = 20.5) and 64.2 (SD = 19.8) on the QLQ-C30 Functional and QLQ-CR29 Functional scales, respectively, reflecting generally good functioning across various life domains.

**Table 3:** Mean and standard deviation (SD) of quality-of-life measures for colorectal cancer patients, (N = 169).

QLQ	Mean	SD	Median	IQR
QLQ-C30 – Global Scale	70.364	18.542	66.67	25.00
QLQ-C30 – Functional Scale	80.565	20.541	86.67	26.67
QLQ-C30 – Symptom Scale	16.406	18.526	12.12	21.21
QLQ-CR29 – Functional Scale	64.215	19.840	71.43	23.81
QLQ-CR29 – Symptom Scale	47.828	10.390	45.68	11.11

**Figure 4:**
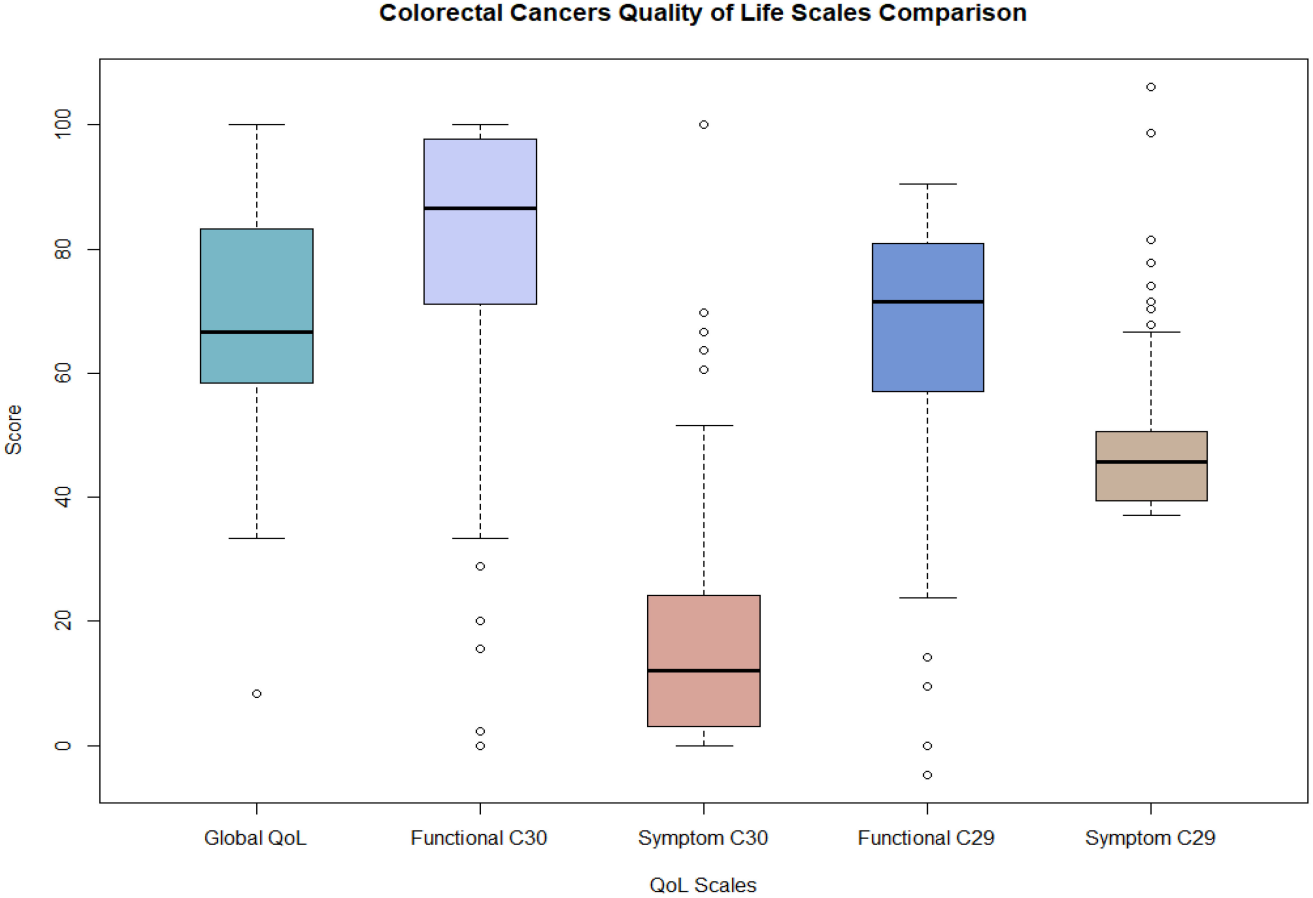
Colorectal cancer quality of life scales box plot, (N = 169).

Conversely, symptom burden appeared to be low, as evidenced by mean scores of 16.4 (SD = 18.5) and 47.8 (SD = 10.4) on the QLQ-C30 Symptom and QLQ-CR29 Symptom scales, respectively. These findings suggest that the colorectal cancer patients in this cohort maintained a relatively good quality of life with minimal symptomatology.

**Table 4** presents the mean scores and standard deviations of the EORTC QLQ-C30 Global Health Status/Quality of Life (QoL) scale, stratified by age group, gender, and cancer stage. Overall, global QoL scores were relatively consistent across age categories, except for the 55–64 age group, which reported the highest mean score of 76.27 ± 15.3.

**Table 4:** QLQ-C30 global quality of life scale scores averages and standard deviations based on age, gender and stage of cancer for colorectal cancer patients in Qatar, (N = 169).

Variable	Global quality of life scale mean	Standard deviation
	*(M)*	*(SD)*
Age		
25-34	64.58	12.5
35-44	68.39	18.7
45-54	69.12	20.9
55-64	76.27	15.3
65 and above	67.09	18.3
Gender		
Male	70.78	19.9
Female	69.94	17.2
Stage of cancer		
Stage 1	75	17.9
Stage 2	67.7	20
Stage 3	70.8	17.9
Stage 4	70.2	18.7

Gender-based comparisons revealed minimal variation, with males reporting a mean global QoL score of 70.78 ± 19.9 and females reporting 69.94 ± 17.2, indicating comparable perceived quality of life between sexes.

Regarding cancer stage, patients with stage I disease reported the highest global QoL score (75.0 ± 17.9), while those with stage II had the lowest (67.7 ± 20.0). Patients diagnosed with stage III and IV colorectal cancer reported similar global QoL scores of 70.8 ± 17.9 and 70.2 ± 18.7, respectively, suggesting that advanced disease stage did not markedly diminish perceived overall quality of life in this cohort.

**Table 5** presents the mean scores, and standard deviations of the quality-of-life (QoL) subscales derived from the EORTC QLQ-C30 and QLQ-CR29 questionnaires among colorectal cancer patients in Qatar. The findings suggest a generally favorable quality of life, with a mean Global Health Status/QoL score (QLQ-C30) of 70.36 ± 18.5, reflecting a high overall perceived QoL.

**Table 5:** The frequency distribution of the QLQ-C30 and QLQ-CR29 subscales averages and standard deviations among colorectal cancer patients in Qatar during 2023, (N = 169).

Tool	Scales	Subscales	Mean	Standard deviation
			*(M)*	*(SD)*
QLQ-C30	Global	Global QoL	70.36	18.54
	Functional Scale	Physical functioning	80.71	24.99
		Role functioning	81.95	28.49
		Emotional functioning	80.08	26.82
		Cognitive functioning	83.73	23
		Social functioning	76.63	30.93
	Symptom Scale	Fatigue	23.93	26.78
		Nausea and vomiting	6.71	18.03
		Pain	15.98	27.72
		Dyspnea	13.41	26.81
		Insomnia	18.54	30.4
		Appetite loss	10.06	23.52
		Constipation	13.41	26.56
		Diarrhea	9.27	22.41
		Financial difficulties	28.99	40.43
QLQ–CR29	Symptom Scale	Urinary frequency	20.32	27.55
		Urinary incontinence	6.11	20.14
		Dysuria	7.69	23
		Abdominal pain	12.82	26.97
		Buttock pain	10.45	24.18
		Bloating	17.95	28.64
		Blood and mucus in stool	5.13	16.67
		Dry mouth	15.38	29.32
		Hair loss	10.45	24.45
		Taste	9.27	24.39
		Flatulence	20.36	27.34
		Fecal incontinence	6.15	18.11
		Sore skin	10.91	23.78
		Stool frequency	15.08	23.09
		Embarrassment	13.49	26.37
		Stoma care problems	20.83	29.18
		Impotence	19.11	32.3
		Dyspareunia	11.25	27.01
	Functional Scale	Anxiety	71.79	37.27
		Weight	82.25	31.5
		Body Image	85.93	21.87
		Sexual interest- male	58.94	39.99
		Sexual interest- female	82.92	27.04

In the QLQ-C30 functional domains, higher scores indicate better functioning and, therefore, better QoL. Cognitive functioning demonstrated the highest mean score (83.7 ± 23.0), while social functioning was the lowest among the functional scales (76.6 ± 30.9), though still relatively high.

Conversely, higher scores in symptom domains reflect greater symptom burden and, hence, poorer QoL. Within the QLQ-C30 symptom scales, financial difficulties were reported as the most burdensome symptoms (28.99 ± 40.4), followed by fatigue (23.9 ± 26.8), whereas nausea and vomiting were the least reported symptoms (6.7 ± 18.0).

The QLQ-CR29 subscales further delineate disease-specific functional and symptom-related QoL aspects. Among functional domains, body image received the highest score (85.9 ± 21.9), followed by sexual interest in females (82.9 ± 27.0) and weight perception (82.3 ± 31.5), indicating positive self-perception in these areas. In contrast, sexual interest among males showed the lowest functional score (58.9 ± 39.99), suggesting a greater impact on sexual functioning in this subgroup.

Among the QLQ-CR29 symptom domains, stoma-related problems were the most reported (20.83 ± 29.2), followed closely by flatulence (20.4 ± 27.3) and urinary frequency (20.3 ± 27.6).

To examine the relationship between the six qualities of life scales and the study factors, a parametric model, i.e., multivariate multiple linear regression. However, the parametric regression model’s assumptions for normality were not met, hence, fitting a parametric regression model to study the effect of the sociodemographic and the clinical variables on patients’ quality of life isn’t appropriate (20). Alternatively, five nonparametric bootstrap regression models were fitted. Non-parametric regression overcomes the normality condition since it makes no assumptions about the population distribution (21).

**Table 6** presents the association between the Global Quality of Life (QoL) scale of colorectal cancer patients in Qatar and various sociodemographic and clinical factors included in the study. In this scale, higher scores reflect better overall quality of life.

**Table 6:** Non-parametric bootstrapped regression results of global Quality of Life scale scores against the sociodemographic and the clinical variables, (N = 169).

Variables	Beta ^a^	Std-error	P-value	Confidence interval	
Age	0.121	0.161	0.45	-0.193	0.436
Gender					
Male (Ref)					
Female	0.873	3.424	0.799	-5.837	7.584
Nationality					
Non-Qatari (Ref)					
Qatari	-2.477	4.333	0.568	-10.97	6.016
Marital status					
Not Married (Ref)					
Married	-2.228	4.878	0.648	-11.789	7.333
Education					
Before Secondary School (Ref)	-6.497	3.316	0.05	-12.996	0.001
Secondary School or Higher					
Employment status					
Not Employed (Ref)					
Employed	3.693	3.878	0.341	-3.908	11.295
Monthly income					
Insufficient (Ref)					
Partially Sufficient	-5.068	4.007	0.206	-12.921	2.786
Sufficient	3.847	3.909	0.325	-3.814	11.509
Comorbidities					
No (Ref)					
Yes	-3.293	3.265	0.313	-9.693	3.106
Smoking					
Non-Smoker (Ref)					
Smoker	7.502	4.856	0.122	-2.016	17.019
Alcohol					
No (Ref)					
Yes	-4.689	5.745	0.414	-15.95	6.571
Cancer stage					
Stage 1 (Ref)					
Stage 2	-6.673	6.986	0.339	-20.365	7.019
Stage 3	-6.639	7.981	0.405	-22.282	9.004
Stage 4	-4.555	8.628	0.598	-21.467	12.356
Time to Diagnose	-0.005	0.034	0.889	-0.072	0.063
Metastasis					
No (Ref)					
Yes	-0.647	5.368	0.904	-11.167	9.874
Treatment type					
Mixed - Chemo & Surgery (Ref)					
Other	-4.166	3.965	0.293	-11.936	3.605

Beta ^a^: Bootstrapped Regression Coefficient.

The analysis revealed that none of the examined variables were significantly associated with the Global QoL scale. However, educational level was found to be borderline significant (*p* = 0.05). Specifically, patients with secondary school education or higher reported a mean Global QoL score that was 6.5 points lower than those with elementary education or less (coefficient = –6.497), suggesting a potential trend toward lower perceived quality of life among more educated patients, though this result should be interpreted with caution.

**Table 7** presents the relationship between key sociodemographic and clinical factors and the EORTC QLQ-C30 functional and symptom scale scores, reflecting quality of life among colorectal cancer patients. In this instrument, higher functional scores indicate better quality of life, whereas higher symptom scores reflect poorer quality of life.

**Table 7:** Non-parametric bootstrapped regression results of EORTC QLQ-C30 scale scores against the sociodemographic and the clinical variables, (N = 169).

Variables	Symptom C 30 Scale					Functional C 30 Scale				
	Beta ^a^	Std-error	p-value	Confidence Interval		Beta ^a^	Std-error	p-value	Confidence Interval	
Age	-0.076	0.111	0.496	-0.293	0.142	-0.131	0.139	0.346	-0.404	0.142
Gender										
Male (Ref)										
Female	0.273	2.485	0.913	-4.599	5.144	-3.046	3.089	0.324	-9.101	3.009
Nationality										
Non-Qatari (Ref)										
Qatari	-4.078	2.98	0.171	-9.919	1.762	0.407	3.745	0.913	-6.933	7.748
Marital status										
Not Married (Ref)										
Married	-5.981	3.562	0.093	-12.963	1	0.218	4.315	0.96	-8.239	8.675
Education										
Before Secondary School (Ref)										
Secondary School or Higher	4.511	2.301	0.05	0	9.022	-4.824	2.984	0.106	-10.67	1.025
Employment status										
Not Employed (Ref)										
Employed	-1.696	2.724	0.534	-7.036	3.644	0.857	3.366	0.799	-5.74	7.454
Monthly income										
Insufficient (Ref)										
Partially Sufficient	-1.229	2.895	0.671	-6.902	4.444	6.024	3.585	0.093	-1.002	13.049
Sufficient	-6.859	2.816	0.015*	-12.378	-1.341	12.25	3.49	0.000*	5.41	19.09
Comorbidities										
No (Ref)										
Yes	2.094	2.25	0.352	-2.316	6.504	-2.917	2.782	0.294	-8.369	2.535
Smoking										
Non-Smoker (Ref)										
Smoker	-2.902	3.583	0.418	-9.925	4.121	1.123	4.396	0.798	-7.492	9.738
Alcohol										
No (Ref)										
Yes	-2.351	4.137	0.57	-10.46	5.758	5.069	5.22	0.332	-5.162	15.3
Cancer stage										
Stage 1 (Ref)										
Stage 2	6.384	5.152	0.215	-3.713	16.481	-11.5	6.305	0.068	-23.86	0.852
Stage 3	5.117	5.871	0.383	-6.39	16.624	-5.206	7.188	0.469	-19.29	8.882
Stage 4	9.086	6.274	0.148	-3.212	21.383	-5.243	7.702	0.496	-20.33	9.852
**Time to Diagnose**	-0.027	0.025	0.289	-0.076	0.023	0.061	0.031	0.048*	0.001	0.122
Metastasis										
No (Ref)										
Yes	0.136	3.83	0.972	-7.371	7.642	-3.558	4.686	0.448	-12.74	5.626
Treatment type										
Mixed - Chemo & Surgery (Ref)										
Other	1.662	2.825	0.556	-3.874	7.198	-3.441	3.538	0.331	-10.37	3.493

Beta ^a^: Bootstrapped Regression Coefficient. P< 0.05*.

Analysis of the Functional C30 scale showed that monthly income and time since diagnosis were significantly associated with improved functional outcomes. Patients reporting sufficient income scored 12.5 points higher than those with insufficient income, indicating a notably better functional quality of life. There was no statistically significant difference between patients with insufficient and partially sufficient income. Additionally, for each additional month since diagnosis, functional scores increased by 0.06 points, suggesting a modest but positive association between survival time and functional well-being.

In contrast, findings from the Symptom C30 scale indicated that only income had a statistically significant impact on symptom burden. Patients with sufficient monthly income scored approximately 9 points lower than those with insufficient income, reflecting fewer symptoms and a better quality of life. While not statistically significant at the conventional threshold, educational level approached significance (*p* = 0.05). Patients with secondary school education or higher scored 4.5 points higher on the symptom scale compared to those with elementary education or less, suggesting a trend toward increased symptom burden in the more educated group.

**Table 8** presents the associations between patient characteristics and the EORTC QLQ-CR29 functional and symptom scale scores, which reflect quality of life in colorectal cancer patients. Higher scores on the functional scale denote better quality of life, while higher scores on the symptom scale indicate poorer quality of life.

**Table 8:** Non-parametric bootstrapped regression results of EORTC QLQ-CR29 scale scores against the sociodemographic and the clinical variables, (N = 169).

	Symptom CR 29 scale					Functional CR29 scale				
Variables	Beta ^a^	Std-error	P-value	Confidence interval		Beta ^a^	Std-error	P-value	Confidence interval	
Age	-0.003	0.061	0.966	-0.123	0.117	0.418	0.118	0.000*	0.187	0.648
Gender										
Male (Ref)										
Female	0.636	1.373	0.643	-2.055	3.327	1.572	2.618	0.548	-3.559	6.703
Nationality										
Non-Qatari (Ref)										
Qatari	-2.002	1.626	0.218	-5.189	1.186	5.843	3.139	0.063	-0.309	11.996
Marital Status										
Not Married (Ref)										
Married	-0.53	1.963	0.787	-4.377	3.316	3.889	3.695	0.292	-3.352	11.131
Education										
Before Secondary School (Ref)										
Secondary School or Higher	1.739	1.277	0.173	-0.763	4.242	-4.951	2.47	0.045*	-9.792	-0.111
Employment Status										
Not Employed (Ref)										
Employed	1.179	1.472	0.423	-1.707	4.065	-3.143	2.819	0.265	-8.669	2.382
Monthly Income										
Insufficient (Ref)										
Partially Sufficient	-0.033	1.574	0.983	-3.118	3.052	-1.462	3.029	0.629	-7.4	4.475
Sufficient	-2.81	1.556	0.071	-5.861	0.24	2.815	3.013	0.35	-3.091	8.72
Comorbidities										
No (Ref)										
Yes	2.067	1.213	0.088	-0.311	4.445	-3.327	2.342	0.155	-7.917	1.263
Smoking										
Non-Smoker (Ref)										
Smoker	-0.099	1.924	0.959	-3.87	3.672	3.007	3.721	0.419	-4.286	10.3
Alcohol										
No (Ref)										
Yes	-1.096	2.178	0.615	-5.364	3.173	-11.23	4.326	0.009*	-19.71	-2.759
Cancer Stage										
Stage 1 (Ref)										
Stage 2	2.805	2.707	0.3	-2.501	8.111	0.953	5.381	0.859	-9.594	9.731
Stage 3	-0.708	3.092	0.819	-6.769	5.353	-2.175	6.074	0.72	-14.08	12.635
Stage 4	-0.505	3.318	0.879	-7.008	5.998	0.009	6.442	0.999	-12.61	0.089
**Time to Diagnose**	-0.021	0.013	0.114	-0.047	0.005	0.038	0.026	0.142	-0.013	0.122
Metastasis										
No (Ref)										
Yes	2.183	2.096	0.298	-1.925	6.292	0.133	4.047	0.974	-7.8	8.065
Treatment Type										
Mixed - Chemo & Surgery (Ref)										
Other	-0.703	1.549	0.65	-3.738	2.333	1.58	2.931	0.59	-4.165	7.326

P< 0.05*.

Analysis of the functional scale revealed significant associations with age at diagnosis, educational level, and alcohol consumption. Specifically, for each one-year increase in age at diagnosis, the functional score increased by 0.42 points (indicating improved quality of life). Patients with secondary school education or higher scored, on average, 5 points lower than those with elementary education or less, indicating a lower perceived functional quality of life. Additionally, alcohol consumers scored 11.2 points lower than non-drinkers, suggesting a significantly poorer functional status among those who consume alcohol.

In contrast, analysis of the symptom scale found no variables with statistically significant associations. However, monthly income and the presence of comorbidities were found to be borderline significant. Patients reporting sufficient income tended to have lower symptom scores, indicating a better quality of life, whereas those with comorbid conditions had higher symptom scores, suggesting greater symptom burden and reduced quality of life.

## 4 Discussion

This study assessed the quality of life (QoL) and its predictors among colorectal cancer (CRC) patients in Qatar during 2023 using the EORTC QLQ-C30 and QLQ-CR29 instruments. Our findings revealed that CRC patients in Qatar reported a relatively good overall QoL, with a mean Global Health Status score of 70.4 (SD = 18.5). This aligns with findings from Sweden by Sjövall A et al. (2023), who also used the QLQ-C30 and CR29 tools and reported similar QoL patterns, although 39% of their patients had clinically impaired global QoL (22). Likewise, Qedair et al. (2021) in Saudi Arabia reported a lower mean global health score of 63.9 (SD = 24.8), suggesting a comparatively better QoL in our study cohort (23). However, these findings were contradicted with Abu-Helalah et al. study as the participants reported lower mean global health score 56.9 ± 31.3 (24), and with findings from Refay et al. study in Egypt 2024 among 132 patients where the average global health score was 41.4(95% CI 37.8 to 44.98) (25).

In Colombia 2024, a cross-sectional study conducted by Flórez et al., found that patients with stable financial conditions and strong social support networks reported higher global QoL, consistent with our findings linking income sufficiency with higher QoL (26). Belaid et al. (2023) also found favorable QoL scores among Tunisian CRC patients, particularly among those with early-stage disease and adequate psychosocial support, which echoes our own patients’ functional well-being (27).

Our participants demonstrated strong functional health, with mean scores of 80.6 on the Functional C30 scale and 64.2 on the Functional CR29 scale. These findings are consistent with results from Flórez et al. (2024), who reported relatively high functional scores among Colombian CRC patients with adequate healthcare access and family support (26), and Belaid et al. (2023), who observed similar functional outcomes among Tunisian patients, particularly those receiving early intervention and multidisciplinary care (27). Similarly, Drury et al. (2020) emphasized that continuity of care and effective communication with healthcare providers were associated with enhanced physical and role functioning, suggesting that structured care pathways may contribute to the high functional scores observed in our cohort (28).

The Symptom burden in our cohort was low to moderate, with a mean Symptom C30 score of 16.4 and Symptom CR29 score of 47.8, indicating relatively fewer physical complaints. Notably, within the QLQ-C30 symptom scales, financial difficulties emerged as the most burdensome symptom (mean = 28.99, SD = 40.4), followed by fatigue (mean = 23.9, SD = 26.8), whereas nausea and vomiting were the least reported (mean = 6.7, SD = 18.0). This pattern is comparable to findings by Turnbull, J. D et al. (2025), and by Abu-Helalah et al. (2022) who also reported fatigue and financial problems as common concerns among CRC patients (24, 29). Similar symptoms have been documented by Arndt et al. (2004) and other European studies, where fatigue and financial hardship were ranked among the top distressing symptoms, often exceeding pain or gastrointestinal complaints (29).

The QLQ-CR29 subscale offered further insights into disease-specific aspects of QoL. Our participants reported high functional scores in body image (85.9), female sexual interest (82.9), and weight perception (82.3), indicating positive self-perception. In contrast, male sexual interest was the lowest-scoring domain (58.9), suggesting notable sexual dysfunction among men. Kim SE et al. (2015) and Chie et al. (2012) similarly reported gender disparities in sexual functioning post-treatment, with men experiencing greater impairment (30, 31). Belaid et al. (2023) reported similar sexual difficulties, especially among male survivors and those with stoma or rectal tumors (27). However, findings from Sweden were contradictory, as women showed lesser sexual interest compared to the male (3.5% *vs* 19.5%), respectively (22). And from Portugal Marchewczyk P et al. with mean scores of 38.4 (95% CI, 32.7–44.2) for males and 16.6 (95% CI, 10.8–22.4) for females (31).

Among the symptom domains of the CR29, stoma-related problems (20.83), flatulence (20.4), and urinary frequency (20.3) were most reported among our participants. These findings align with the Swedish data from Sjövall A et al. (2023), as well as with the results of Flyum et al. (2021), whose meta-analysis identified stoma-related concerns, abdominal symptoms, and urinary disturbances as common ongoing issues among CRC patients in the palliative phase (8, 22).

Regarding QoL predictors, our study found sufficient income to be a strong positive determinant of both better functional outcomes and fewer symptoms. This is consistent with the findings of Färkkilä et al. (2014) and Newcomer et al. (2025), who emphasized the role of socioeconomic stability in emotional well-being and QoL (9, 32). Flórez et al. (2024) also noted that financial hardship was a significant driver of lower QoL, particularly among unemployed or uninsured patients (26). Time since diagnosis was positively associated with better functional scores, suggesting adaptive coping, similar to observations by Newcomer et al. (2025), and Abu-Helalah et al. (2022) (24, 32). Additionally, older age diagnosis was linked to better QoL in our sample, a trend previously identified in studies by Jansen et al. (2010) and Drury et al. (2020), who reported that older patients may exhibit greater resilience and lower symptom sensitivity (5, 28).

Unexpectedly, higher education in our study was associated with slightly lower QoL scores. While this contradicts the positive association seen in Belaid et al. (2023), it aligns with findings from Al-Dahshan et al. (2020), who suggested that more educated patients may have greater expectations and heightened awareness of health status, potentially leading to lower self-rated QoL (27, 33), This may also be attributed to the fact that more educated patients often have greater health literacy and higher expectations regarding their treatment and recovery, making them more critical of their perceived health. Additionally, the increased psychological burden and heightened awareness of symptoms among highly educated individuals may further contribute to slightly lower QoL scores. Alcohol consumption was associated with lower functional scores in our analysis, in line with Fedirko V et al. (2010), who found that alcohol use in CRC patients was linked to increased symptoms and poorer survival outcomes (34). Finally, while most predictors influenced functional and symptom scales, none significantly affected the global QoL score, although education approached the significance threshold. This may reflect the multifactorial and subjective nature of global QoL perception, as highlighted in studies by Jansen et al. (2010), and Flyum et al. (2021) (5, 8).

In our study, patients with advanced colorectal cancer (Stage III–IV) reported overall quality of life (QoL) comparable to those with early-stage disease. International evidence on this association remains inconsistent. For example, a study from the United States by Feizpour et al. (2023) demonstrated that Stage IV patients experienced poorer symptom-related QoL, suggesting that metastatic burden may negatively affect specific QoL domains (35). Conversely, a Swedish cohort study by Sjövall et al. (2023) reported clinically relevant impairment in global QoL among a substantial proportion of early-stage (I–III) patients one year after surgery, indicating that early stage does not necessarily confer preserved QoL (22).

Importantly, these discrepancies may be explained by the prognostic role of tumor characteristics beyond conventional TNM staging. As highlighted in recent literature, tumor grade, tumor budding, lympho-vascular and perineural invasion, and molecular features such as microsatellite instability and key oncogenic mutations remain clinically relevant prognostic factors in colorectal cancer, even in the era of personalized medicine (36). These tumor-specific characteristics influence disease aggressiveness, treatment strategies, and long-term outcomes, and may contribute to heterogeneous QoL experiences among patients with similar clinical stages. Consequently, comparable global QoL scores may be observed despite differences in stage, reflecting the complex interplay between tumor biology, treatment effects, and patient adaptation (36).

## 5 Study strengths and challenges

### 5.1 Strengths

We used standardized assessment tools, such as the European Organization for Research and Treatment of Cancer Core Questionnaire (EORTC QLQ-C30) and its CRC-specific module (QLQ-CR29). We also had well-trained data collectors who contributed to data quality and accuracy, enhancing the findings’ overall reliability.

### 5.2 Challenges

As with any cross-sectional study, there is a potential for recall and selection bias. Conducting a telephone survey may introduce biases since individuals who do not answer or choose not to participate could differ from those who do participate in terms of both characteristics and symptoms.

## 6 Conclusion and recommendations

In this study of colorectal cancer patients in Qatar, most were older adults, non-Qatari, and diagnosed at advanced stages, with adenocarcinoma predominating and the sigmoid colon as the most frequent tumor site. Despite late-stage presentation, patients reported relatively high global quality of life, with favorable functional outcomes and low symptom burden. Functional QoL was higher among patients with sufficient income and longer time since diagnosis, and lower among those with higher education or alcohol use. Comorbidities increased symptom burden.

These findings emphasize the importance of integrating psychosocial and financial support, lifestyle counseling, and routine QoL monitoring into standard care to identify at-risk patients and optimize outcomes. Future studies should evaluate the impact of targeted supportive interventions on long-term QoL.

## Data Availability

The raw data supporting the conclusions of this article will be made available by the authors, without undue reservation.
